# Practice patterns, attitudes, and knowledge among clinicians regarding hyperthermic intraperitoneal chemotherapy and pressurized intraperitoneal aerosol chemotherapy: a national survey by Indian society of peritoneal surface malignancies (ISPSM)

**DOI:** 10.1515/pp-2020-0120

**Published:** 2020-08-31

**Authors:** Sampige Prasanna Somashekhar, Kumar C. Rohit, S. V. S. Deo, Kyatsandra Rajagopal Ashwin

**Affiliations:** Department of Surgical Oncology, Manipal Comprehensive Cancer Center, Manipal Hospital, Bangalore, India; All India Institute of Medical Sciences, New Delhi, India

**Keywords:** cytoreductive surgery (CRS), hyperthermic intraperitoneal chemotherapy (HIPEC), peritoneal surface malignancy (PSM), pressurized intraperitoneal aerosol chemotherapy (PIPAC), survey

## Abstract

**Objectives:**

Perception of cytoreductive surgery (CRS), hyperthermic intraperitoneal chemotherapy (HIPEC), and pressurized intraperitoneal aerosol chemotherapy (PIPAC) for treating peritoneal surface malignancies (PSM) differ widely among physicians.

**Methods:**

This on-site survey performed during a major oncology congress in 2019 evaluated the current opinion, perceptions, knowledge and practice of HIPEC and PIPAC among oncologists in India.

**Results:**

There were 147 respondents (gynecologists (30%), surgical oncologists and gastrointestinal surgeons (64%), and medical oncologists (6%)). Whereas most respondents considered CRS and HIPEC an appropriate therapeutic option, 25% would not recommend CRS and HIPEC. The main barriers to referral to an expert center were inaccessibility to such a center (37.8%), non-inclusion of CRS and HIPEC in clinical practice guidelines (32.4%), and a high morbidity/mortality (21.6%). Variations were found in the various practice patterns of CRS/HIPEC like eligibility criteria, HIPEC protocols and safety measures. Although PIPAC awareness as a novel therapeutic option was high, only a limited number of centers offered PIPAC, mainly because of non-access to technology and missing training opportunities (76.2%).

**Conclusions:**

Lack of widespread acceptance, poor accessibility and low utilization presents a significant challenge for HIPEC and PIPAC in India. There is a need to raise the awareness of curative and palliative therapeutic options for PSM. This might be achieved by the creation of expert centers, specialized training curricula and of a new sub-speciality in oncology.

## Introduction

Peritoneal surface malignancies (PSM) represents a special locoregional disease pattern limited to the abdominal cavity and has traditionally been considered a death sentence by the medical fraternity due to the very poor prognosis and dismal survival of 6–12 months [1], [2].

The multimodality treatment of cytoreductive surgery (CRS) and heated intraperitoneal chemotherapy (HIPEC) combines radical surgery with circulation of heated chemotherapy in the peritoneal cavity. This is a therapeutic option showing improved outcomes and quality of life compared to standard systemic chemotherapy for appropriately selected patients with PSM. It has been proposed as a treatment option in patients with peritoneal metastasis of colorectal, ovarian, gastric cancers and sarcomas and as a standard treatment for pseudomyxoma peritonei and peritoneal mesothelioma [3], [4], [5], [6], [7], [8].

Pressurized intraperitoneal aerosol chemotherapy (PIPAC) is a novel technique delivering normothermic chemotherapy into the abdominal cavity by laparoscopy as an aerosol under pressure. This concept seems to enhance the effectiveness of intraperitoneal chemotherapy by taking advantage of the physical properties of gas and pressure by generating an artificial pressure gradient and enhancing tissue uptake and distributing drugs homogeneously within the closed and expanded peritoneal cavity. Recommendations of operative technique, safety checklist and treatment protocols are well established [9], [10], [11], [12].

Although the number of specialist centers for PSM in India is increasing, there seems to be two main challenges. Some clinicians still have nihilistic attitude to this disease and still adopt palliative treatment. While in others, who are aware of curative options there exist a lack of acceptance, utilization and a wide variability in management. It is imperative to evaluate the perceptions and opinions of this complex disease among the oncological fraternity. [7], [8], [13].

The aim of this survey-based study was to evaluate factors influencing referral choices, utilization of CRS and HIPEC, assess current practices and knowledge of the specialists that influence treatment for PSM [14], [15].

## Materials and methods

The survey was submitted to the attendees of the International conference of European Society of Surgical Oncology (ESSO) and Indian Society of Peritoneal Surface Malignancy (ISPSM) at TATA memorial cancer center, Mumbai on April 19th–21st, 2019. There were 228 delegates who attended the annual conference out of which 147 participated in the survey. The participants of the survey were super specialists involved in treating PSM regularly. The survey consisted of an independently developed 33 multiple choice questionnaires. The questionnaire was pilot-tested among the oncologists within our institution for assessment and changes were made based on feedback. It was divided into four parts. The first part had six items to characterize respondents; second part had six items to assess patient presentation, perioperative staging, patient selection and referral patterns. The third part had 12 questions to evaluate the patient eligibility, knowledge, surgical practice, HIPEC practices and safety measures during CRS and HIPEC. The 4th part evaluated the PIPAC related characteristics and responses.

A descriptive statistical analysis was carried out and described quantitative and qualitative data according to means (± standard deviation), medians (range) and percentages. The percentages were calculated over all the responses received for each question.

## Results

**Figure 1:**
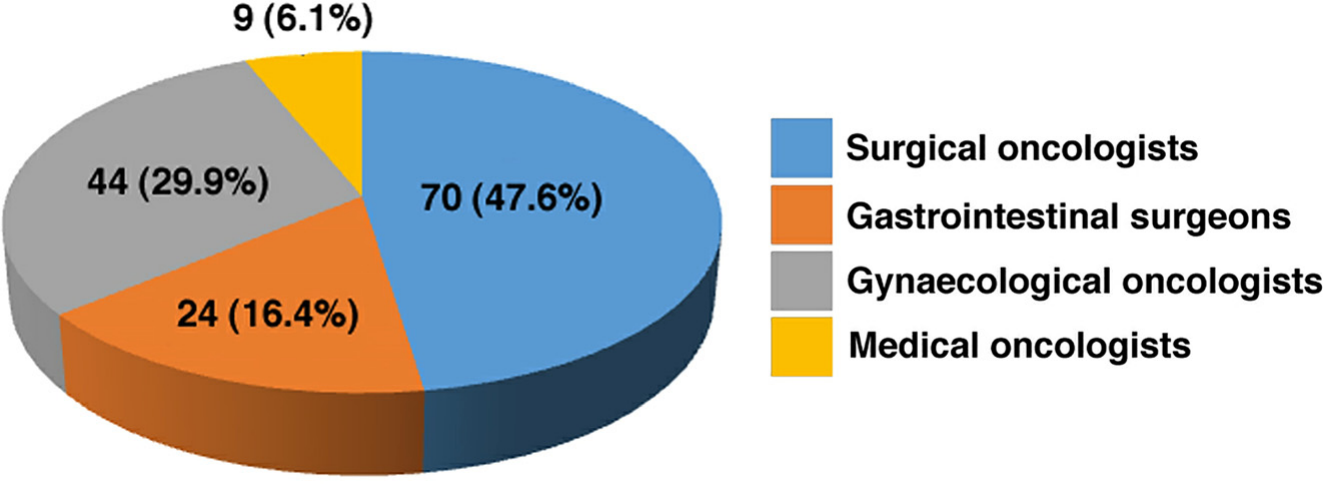
Subspecialty of the survey respondents.

**Figure 2:**
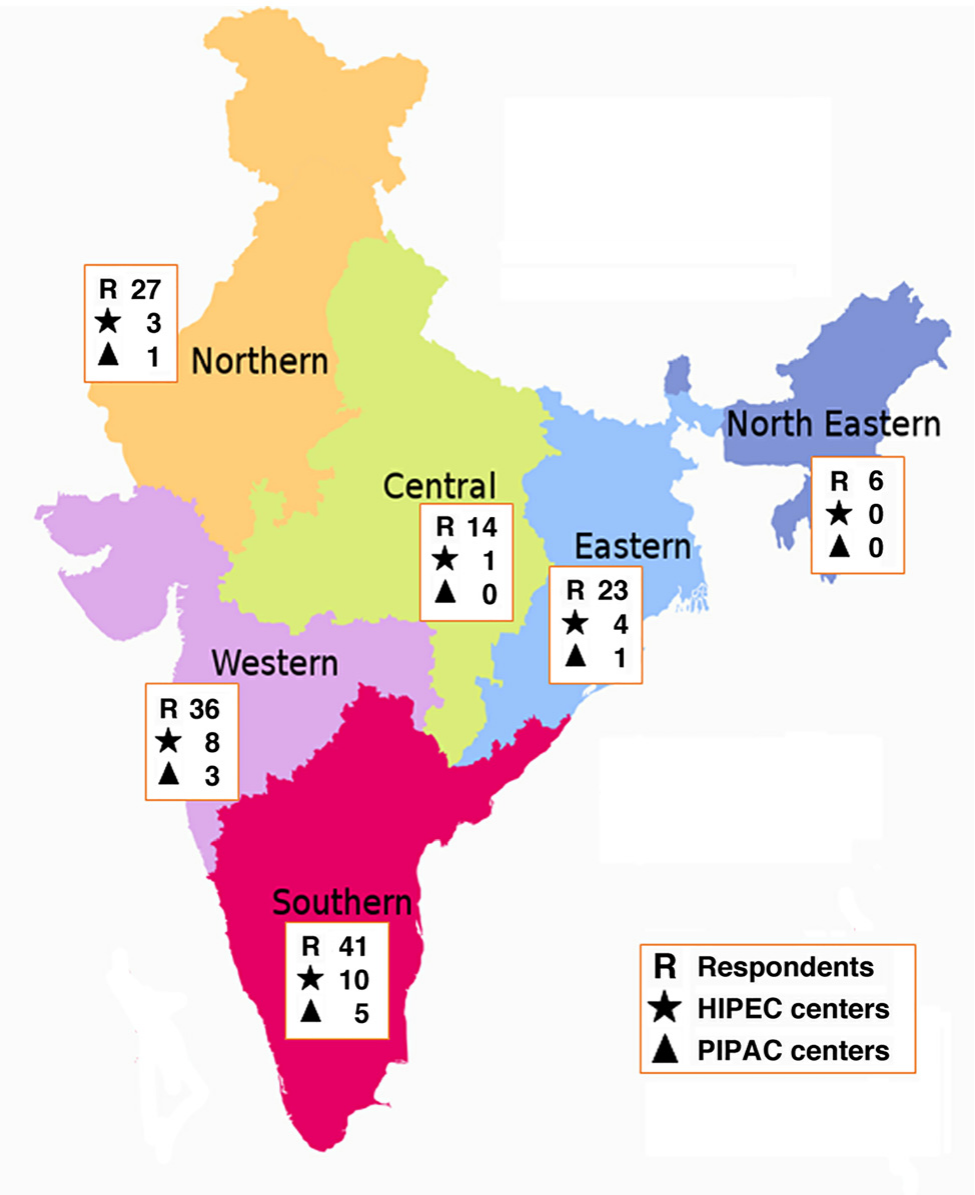
Distribution of respondents, HIPEC and PIPAC centers according to zones.

We compiled and analyzed the results from 147 participants (Figures 1 and 2).

Demographic characteristics of survey respondents regarding PSM are shown in Table 1. Majority of doctors practiced in a medical college (32.6%) or private teaching institute (25.8%). Awareness regarding HIPEC treatment modality was learnt from peers or colleagues in practice (38.1%) or from scientific academic meetings (36%). More than half the doctors (53.7%) had not been involving any PSM specific treatment at the time of survey while 30.6% were working in the department which offered CRS/HIPEC. 25.9% of the respondents were actively involved in offering CRS/HIPEC to their patients and 8.2% also offered PIPAC. Close to 75% of the participants had personally never performed the procedure and only 17.4% of them had access to a surgeon with expertise in CRS and HIPEC.

**Table 1:** Demographic characteristics of survey respondents.

Questions	n=147 (%)
Type of hospital where you practice	
Medical college	48 (32.6%)
Private teaching hospital	38 (25.8%)
Private hospital	61 (41.5%)
Where did you first learn about CRS/HIPEC and PIPAC?	
Training program (residency or fellowship)	21 (14.3%)
Scientific meetings	53 (36%)
From colleagues in practice	56 (38.1%)
Peer-reviewed literature	17 (11.6%)
Does your department offer the following treatment options for PSM? *(multiple answers possible)*	
Hyperthermic intraperitoneal chemotherapy (HIPEC)	45 (30.6%)
Intraperitoneal catheter therapy	23 (15.6%)
Pressurized intraperitoneal aerosol chemotherapy (PIPAC)	12 (8.2%)
None	79 (53.7%)
Have you performed CRS and HIPEC procedures for PSM till now?	
Yes	38 (25.9%)
No	109 (74.1%)
If no, is there a surgeon with expertise in CRS and HIPEC available to treat your patients?	(n=109)
Yes	19 (17.4%)
No	90 (82.6%)

CRS, Cytoreductive surgery; HIPEC, Hyperthermic Intraperitoneal Chemotherapy; PIPAC, Pressurized Intraperitoneal Aerosol Chemotherapy; PSM: Peritoneal surface Malignancy.

Responses to questions regarding presentation characteristics, referral and practice patterns regarding CRS and HIPEC are summarized in Table 2. The most common presentation encountered by Indian physicians in clinical practice is peritoneal carcinomatosis secondary to ovarian cancer (63.2%). 58.5% diagnosed fewer than 10 patients with PSM annually. The diagnostic imaging of choice at presentation was abdominal CT scan (71.4%). Approximately 86% of respondents considered CRS/HIPEC as an appropriate therapeutic option for appendicular cancer (pseudomyxoma peritonei) and 51, 46.2, 66.7% considered it appropriate for ovarian cancer, colon cancer and peritoneal mesothelioma respectively. Interestingly 25% of the doctors surveyed indicated they would not recommend CRS/HIPEC. The most common reason was inaccessibility to an HIPEC expert (37.8%). Other factors impacting the decision not to offer the therapy was lack of level 1 evidence (27%), non-inclusion in National Comprehensive Cancer Network (NCCN) guidelines (32.4%) and associated high morbidity/mortality (21.6%).

**Table 2:** Response to presentation characteristics, referral practice patterns regarding CRS/HIPEC.

Questions	n=147 (%)
What are the common presentations of PSM in your hospital?(*multiple answers possible*)	
Pseudomyxoma	68 (46.2%)
Gastric origin cancer	64 (43.5%)
Peritoneal mesothelioma	26 (17.7%)
Colorectal origin cancer	70 (47.6%)
Ovarian cancer	93 (63.2%)
How many patients with PSM do you see annually?	
<10	86 (58.5%)
10–20	30 (20.4%)
>20	31 (21.1%)
What is your diagnostic imaging of choice for measuring extent of cancer in PSM?	
Abdominal ultrasonography	0
Abdominal CT	105 (71.4%)
Abdominal MRI	27 (18.3%)
Whole body PET CT	15 (10.2%)
Do you discuss the management of your patients in a multidisciplinary tumor board meeting?	
Yes	59 (40.1%)
No	88 (59.9%)
What indications would you consider patients for CRS/ HIPEC as a therapeutic option? *(multiple answers possible)*	
Appendiceal cancer (pseudomyxoma peritonei)	126 (85.7%)
Ovarian cancer	75 (51%)
Colon cancer	68 (46.2%)
Gastric cancer	22 (14.9%)
Peritoneal mesothelioma	98 (66.7%)
Other	7 (4.7%)
None	37 (25.1%)
Select reasons why you have not consider patients for CRS/HIPEC (multiple answers possible)	
Don’t have access to a HIPEC specialist	14 (37.8%)
Evidence to support CRS and HIPEC is insufficient	10 (27%)
The morbidity and mortality of CRS and HIPEC is too high	8 (21.6%)
NCCN guidelines do not completely support use of CRS/HIPEC	12 (32.4%)

PSM, Peritoneal surface Malignancy; CT, Computerized tomography; MRI, Magnetic resonance imaging; PET CT, Positron emission tomography Computerized tomography; CRS, Cytoreductive surgery; HIPEC, Hyperthermic Intraperitoneal Chemotherapy; NCCN, National Comprehensive Cancer Network.

Table 3 summarizes the responses to the knowledge and safety based questions answered by experts who have performed CRS/HIPEC. Thirty- eight of our respondents had performed CRS/HIPEC and were familiar with the procedure. Most of them had started to perform the procedure recently and completed less than 10 procedures, 60.5% had received formal hands on training at a center of excellence. Poor Eastern Cooperative Oncology Group (ECOG) performance status and mesenteric invasion was an absolute contra indication. The other indications included multi organ involvement (92.1%) and frozen pelvis (78.9%). More than 80% found financial implications as an important factor for offering the procedure to the patient. More than 90% of surgeons had access to FDA approved HIPEC machine at their institution. 39.5% of the surgeons equally preferred coliseum and closed method for performing HIPEC. Some of them, 21% used the semi open method too. Most of the surgeons (73.7%) place abdominal drains routinely after HIPEC.

**Table 3:** Pre-operative assessment, patient selection, expertise and safety response.

Questions	n=147 (%)
How many CRS/HIPEC procedures for PSM have you performed till now?	
None	109 (74.1%)
Yes	38 (25.9%)
Below 10	17 (11.5%)
11–30	12 (8.2%)
31–50	4 (2.7%)
Above 50	5 (3.4%)
	n=38 (%)
Have you had any formal training in CRS/HIPEC?	
Yes	23 (60.5%)
No	15 (39.5%)
What factors that prevents you from offering CRS/HIPEC in indicated patients with PSM?	
Old age	5 (13.1%)
ECOG performance status	38 (100%)
Invasion to numerous mesenteries	38 (100%)
Cancer that invades multiple organs (more than 3 organs)	35 (92.1%)
Cancer that invades frozen pelvis	30 (78.9%)
Ureteral stricture	21 (55.3%)
Others (cost)	31 (81.6%)
Drains following CRS and HIPEC	
No	3 (7.8%)
Only for resection anastomosis	7 (18.4%)
Routinely for all	28 (73.7%)
Intraoperative chemotherapy agent used	
Ovarian Cancer	
Cisplatin 90 min	25 (65.8%)
Cisplatin + Doxorubicin/Adriamycin 90 min	8 (21%)
Oxaliplatin + Doxorubicin 90 min	0
No response	5 (13.2%)
Colon Cancer	
Oxaliplatin + 5FU IV 30 min	3 (7.9%)
Mitomycin C 90 min	29 (76.3%)
No response	6 (15.8%)
Gastric Cancer	
Cisplatin 60 min	26 (68.4%)
Oxaliplatin	0
Mitomycin C	0
No response	12 (31.6%)
Mesothelioma	
Cisplatin + Doxorubicin/Adriamycin	15 (39.5%)
Oxaliplatin	8 (21%)
No response	15 (39.5%)
Type of HIPEC machine	
FDA-authorized machine	35 (92.1%)
Non FDA authorized machine/Heart lung machine	3 (7.9%)
The temperature of infusing liquid while performing HIPEC	
Under 40 °C	0
40 °C–41 °C	6 (15.8%)
41 °C–42 °C	23 (60.5%)
≥42 °C	9 (23.7%)
What method of HIPEC do you perform?	
Open method/ Colosseum	15 (39.5%)
Closed method	15 (39.5%)
Semi-open	8 (21%)
Average length of critical care stay	
<24 h	6 (15.8%)
<48 h	30 (78.9%)
>72 h	2 (5.2%)
Average length of hospital stay	
<10 days	12 (31.6%)
10–15 days	22 (57.9%)
>15 days	4 (10.5%)
Average time to start adjuvant chemotherapy following CRS and HIPEC	
<3 weeks	4 (10.5%)
3–6 weeks	32 (84.2%)
>6 weeks	2 (5.2%)
Which of the following occupational safety measures do you follow at your center? *(multiple answers possible)*	
Covering film for HIPEC (3 M Ioban surgical cover)	11 (28.9%)
Smoke extractor	10 (26.3%)
Laminar flow equipped OT	18 (47.4%)
Ground covering for possible cytostatic spillage	10 (26.3%)
High-power filtration masks	22 (57.9%)
Occular protection during HIPEC	10 (26.3%)
Long-sleeve double gloving	25 (65.8%)
Shoe Covers	33 (86.8%)
None	29 (76.3%) 1 (2.6%)

PSM, Peritoneal surface Malignancy; CRS, Cytoreductive surgery; HIPEC, Hyperthermic Intraperitoneal Chemotherapy; ECOG, Eastern Cooperative Oncology Group; FU, Fluorouracil; FDA, Food and Drug Administration; OT, Operation theatre.

Table 4 deals with the responses of the surgeons regarding PIPAC. Out of 147 respondents, only 12 had been trained in performing PIPAC. The most common reason for not performing PIPAC was lack of availability of capnopen and training in India (76.2%). Time interval between each PIPAC procedure was 6 weeks for 91.6% and only one center preferred eight weekly. One fourth of them combined PIPAC with concomitant systemic chemotherapy. Regarding chemotherapy agents, the dosage varied for ovarian cancer, where two centres used the lower dosage of 7.5 mg/m^2^ and 1.5 mg/m^2^, respectively.

**Table 4:** PIPAC survey characteristics and responses.

Questions	n=147
Have you performed PIPAC?	
Yes	12 (8.2%)
No	135 (92.8%)
Select reasons why you have not consider patients for PIPAC *(multiple answers possible)*	
Lack of training	112 (76.2%)
Evidence to support is insufficient	12 (8.8%)
NCCN guidelines do not completely support use PIPAC	23 (17%)
	n=12 (%)
How many patients have been treated with PIPAC by you?	
<10	5 (21.6%)
10–25	3 (25%)
25–50	3 (25%)
≥50	1 (8.4%)
What are the peritoneal malignancies you have treated by PIPAC? *(multiple answers possible)*	
Gastric	7 (58.3%)
Colorectal	10 (83.3%)
Ovarian	12 (100%)
Appendix	5 (41.6%)
Peritoneal mesothelioma	2 (16.6%)
What is the mean time between each sequentially performed PIPAC in your institution?	
4 weeks	0
6 weeks	11 (91.6%)
8 weeks	1 (8.4%)
What is the maximal number of PIPAC procedures that you have performed for one patient?	
2 PIPAC procedures	2 (16.6%)
3 PIPAC procedures	6 (50%)
4 PIPAC procedures	1 (8.4%)
5 PIPAC procedures	3 (25%)
Do you combine PIPAC with concurrent systemic chemotherapy?	
Yes	3 (25%)
No	9 (75%)
What radiological evaluation is preferred before or after PIPAC?	
CECT	8 (66.7%)
MRI	4 (33.3%)
PET	0
What type chemotherapy and dose do you use for each pathology?	
Colorectal	
Oxaliplatin 92 mg/m^2^	12 (100%)
Other	0
Ovary	
Cisplatin 10 mg/m^2^ + Doxorubicin 1.5 mg/m^2^	10 (83.3%)
Cisplatin 7.5 mg/m^2^ + Doxorubicin 1.5 mg/m^2^	2 (16.7%)
Gastric/Mesothelioma	
Cisplatin 7.5 mg/m^2^ + Doxorubicin 1.5 mg/m^2^	7 (58.3%)
Other	0

PIPAC, Pressurized Intraperitoneal Aerosol Chemotherapy; NCCN, National Comprehensive Cancer Network; CECT, Contrast enhanced Computerized tomography; MRI, Magnetic resonance imaging; PET CT, Positron emission tomography Computerized tomography.

## Discussion

The ISPSM consists of over 250 members from various parts of India involved with treatment of peritoneal cancer with focus on research and education of PSM. This is the first study evaluating the perceptions, knowledge and practice regarding PSM of clinicians practicing in premier cancer centers of India.

### Current scenario

There is differing approaches to PSM in India at present. Limited adoption and access of CRS/HIPEC for patients underlines the scepticism among the clinicians about its role and efficacy despite mounting evidence. Poor knowledge of procedure and benefit is one of the most important reasons for underutilization [13], [16], [17]. This procedure is technically challenging with high morbidity and mortality, needing an institutional setup with well-equipped OT, anaesthetic and intensive care departments. Numerous studies have demonstrated a consistent relationship between high volume centres and improved long term survival after cancer surgery [18], [19]. At present in India, management of peritoneal carcinomatosis is restricted to selected specialized centres [20]. Most responders preferred abdominal CT scan as preferred method of pre-operative staging. Despite its widespread use internationally, CRS with HIPEC continues to be perceived by the oncologists in India as experimental despite good evidence [3], [4], [5], [6], [7], [21], [22].

### Acceptance and barriers

Overall, 75% of respondents regarded CRS + HIPEC as therapeutically effective, 25% responded that they are not fully convinced and may not refer or offer their patients this option. The main barriers influencing treatment choices ranged from lack of inclusion in clinical practice guidelines, high morbidity/mortality to lack of training and inaccessibility to an HIPEC expert. Interestingly, many respondents indicated that a change of NCCN guidelines may influence their decision to consider it as standard of care. Studies showed lack of familiarity with the results of CRS/HIPEC, both in terms of survival, for various pathologies and morbidity and mortality, would be one of the reasons for poor adoption. These gaps in knowledge and lack of awareness needs to urgently bridged [17], [23], [24]. Few international guidelines have been created to optimize the benefits and minimize the adverse events for patients with colorectal cancer. Similar protocols or guidelines are needed for other pathologies and for specific populations [25], [26], [27], [28], [29].

### Treatment and safety practices

Our study indicated that diffuse mesenteric invasion and poor ECOG score are the most crucial factors impacting the treatment decision for PSM. More than three fourth of the respondents also indicated multi organ involvement and frozen pelvis as the second and third factors, respectively, for eligibility against the use of CRS with HIPEC. Studies have reported that surgeons practicing in high-volume hospitals produce favourable oncologic outcomes and reduced adverse events [29], [30], [31]. As this procedure is technically challenging with high morbidity and mortality, clinical practices for perioperative management has been developed [32], [33]. Inconsistencies were found in the various technical practice patterns of HIPEC like method of HIPEC, selection of cytostatic agents, infusion temperature and safety measures applied which may significantly impact the clinical outcomes. Similar observation was made by other studies too and concluded need for standardization of procedures [34], [35]. The current practices regarding usage of drains, ICU stay, hospital stay and time for starting adjuvant chemotherapy also varied. The use of specialized protective equipment and safety protocols are common but widely variable among the centers. Some practices were widespread, such as long-sleeve double gloving, covering floor for cytostatic spillage, shoe covers and high-power filtration masks while laminar flow equipped OT, some evacuators and ocular protection was uncommon [36], [37].

### Scenario of PIPAC

The application of PIPAC as new treatment approach is performed only at a handful of centers in India. More than 75% of the participants stated the reason for not performing PIPAC was lack of training in India. A well-structured certification training course in India would help to increase the number patients undergoing this novel procedure. The survey on PIPAC indicates that indications, technical aspects and treatment regimens are uniform. This is probably explained by a standardized training by International Society for the Study of Pleura and Peritoneum (ISSPP) to become a PIPAC surgeon. Evaluation of new modalities like intraperitoneal immunotherapy or pressurized intraperitoneal chemotherapy as neoadjuvant therapy or as curative is underway in order to expand the patient selection and improved outcomes [38], [39], [40].

The limitations of this study include moderate sample size and heterogeneity of participants that may not represent the “real-life” picture. Higher rate of “expert” responders might lead to selection bias. Also, PSM encompasses different origins of PSM requiring different treatment approaches and grouping them together may be over simplification of a complex disease.

## Conclusions

The study demonstrates three main findings.

First, research is needed develop new treatment options for patients with co morbidities and poor performance status, border line indications and palliative patients.

Second, patient referral to HIPEC centers is underutilized due to lack of acceptance, adoption and awareness among the medical fraternity. This presents a significant challenge for CRS/HIPEC and strategies are needed to educate and provide also familiarize the clinicians with the procedure by providing surgical training to consultants/residents. Standardization of HIPEC protocols is necessary among the specialists who are already performing the procedure to improve oncological outcomes while decreasing morbidity and mortality.

Third, PIPAC had a high awareness as a novel therapeutic option but the main reason for underutilization of PIPAC in huge country like India was lack of certified training which can easily be solved by setting up centres of excellence with regular structured training courses.

Our study emphasizes a need to raise the awareness of PSM as a specialized branch in oncology, to encourage prospective multicentric studies and to publish protocols for patients based on the best available evidence and consensus among the experts.
